# Peripheral administration of lactate produces antidepressant-like effects

**DOI:** 10.1038/mp.2016.179

**Published:** 2016-10-18

**Authors:** A Carrard, M Elsayed, M Margineanu, B Boury-Jamot, L Fragnière, E M Meylan, J-M Petit, H Fiumelli, P J Magistretti, J-L Martin

**Affiliations:** 1 Center for Psychiatric Neurosciences, Department of Psychiatry, Lausanne University Hospital, Lausanne, Switzerland; 2 Brain Mind Institute, Ecole Polytechnique Fédérale de Lausanne, Lausanne, Switzerland; 3 King Abdullah University of Science and Technology (KAUST), BESE Division, Thuwal, Saudi Arabia

## Abstract

In addition to its role as metabolic substrate that can sustain neuronal function and viability, emerging evidence supports a role for l-lactate as an intercellular signaling molecule involved in synaptic plasticity. Clinical and basic research studies have shown that major depression and chronic stress are associated with alterations in structural and functional plasticity. These findings led us to investigate the role of l-lactate as a potential novel antidepressant. Here we show that peripheral administration of l-lactate produces antidepressant-like effects in different animal models of depression that respond to acute and chronic antidepressant treatment. The antidepressant-like effects of l-lactate are associated with increases in hippocampal lactate levels and with changes in the expression of target genes involved in serotonin receptor trafficking, astrocyte functions, neurogenesis, nitric oxide synthesis and cAMP signaling. Further elucidation of the mechanisms underlying the antidepressant effects of l-lactate may help to identify novel therapeutic targets for the treatment of depression.

## Introduction

Over the past years, evidence has accumulated indicating that glial cells are involved in the pathophysiology of major depression. In particular, reductions in the number and density of glial cells have been observed in different frontolimbic brain regions of depressed patients.^1^ Decreases in glial cell density are accompanied by changes in the expression of several astrocytic markers in frontolimbic cortical regions and subcortical brain areas including the hippocampus of depressed patients, suggesting that astrocyte dysfunction contributes to the pathogenesis of major depressive disorder.^1, 2^

Astrocytes are support cells necessary to ensure neuronal functioning and viability. In this context, astrocytes are involved in essential brain mechanisms and functions including energy metabolism, K^+^ buffering, neurotransmitter recycling, neurogenesis, neuronal plasticity and synaptic transmission.^3, 4^ With regard to energy metabolism, astrocytes have a central role in brain energy production, delivery, utilization and storage. In particular, astrocytes respond to glutamatergic activation by increasing the rate of glucose utilization and the release of lactate,^5^ a metabolic substrate that can support neuronal energy demands. Another metabolic feature of astrocytes with respect to glucose metabolism is that they are the only brain cell type capable of storing glucose as glycogen. Of particular relevance to depression, astrocyte glycogen levels are regulated by noradrenaline and serotonin.^6^ In addition to fulfilling the metabolic needs of astrocytes,^7^ astrocytic glycogen breakdown typically results in the production and release of lactate,^7^ which can sustain neuronal function and viability. Taken together, these findings establish that both glycogen mobilization and increased glycolysis lead to the production and release of lactate by astrocytes, highlighting the important role of this monocarboxylate in brain energetics.

In addition to its role as a neuronal energy substrate, an increasing number of studies indicate that lactate fulfills a signaling role in the brain (for review see Mosienko *et al.*^8^). For instance, studies have shown that the transfer of lactate from astrocytes to neurons is necessary for long-term memory formation^9^ and for cocaine-induced long-lasting behavioral effects.^10^ Interestingly, the role of lactate in these synaptic plasticity mechanisms is independent of its function as an energy source.

Analysis of the molecular mechanisms underlying the role of lactate in synaptic plasticity has revealed that l-lactate increases the expression of plasticity-related genes including *Arc*, *c-Fos*, *Zif268* and *BDNF* in cortical neurons.^11^ Interestingly, evidence indicates that these synaptic plasticity genes are involved in the pathophysiology and treatment of depression. For instance, the expression of *Arc* and *Zif268* is decreased in the prefrontal cortex of depressed subjects and in the medial prefrontal cortex of mice subjected to chronic social defeat stress.^12^ In addition, the expression of *Zif268* and *c-fos* is regulated by different classes of antidepressants in several brain areas.^13, 14^ Post-mortem analysis of brain-derived neurotrophic factor expression has shown increased levels in the rodent hippocampus and in the hippocampus of depressed subjects following antidepressant administration.^15, 16^

Studies in humans and animal models have shown that depression and chronic stress are associated with alterations in synaptic plasticity that are characterized by a decreased number of axospinous synapses and by a reduced expression of synapse-related genes in the prefrontal cortex and hippocampus.^17, 18^ Growing evidence also indicates that reversal of synaptic deficits by antidepressants involves enhanced expression of plasticity-related genes.^17^ Collectively, these observations led us to hypothesize that, by increasing the expression of plasticity-related genes, l-lactate may produce antidepressant-like effects.

The aim of this study was to examine the effects of peripheral l-lactate administration on depressive-like behavior. Here we show that acute and chronic peripheral administration of l-lactate produces antidepressant-like effects. At the cellular level, peripheral l-lactate administration increases hippocampal extracellular l-lactate levels and regulates downstream signaling molecules and target genes that may contribute to its antidepressant action.

## Materials and methods

Methods not described here can be found in Supplementary Information.

### Forced swim test

The forced swim test (FST) was performed as described previously.^19^ Briefly, C57Bl/6 mice were placed in a 5 L cylindrical container filled to a depth of 15 cm with water (23–25 °C). A 10 min swim test session was videotaped, and time spent immobile (defined as minimal movements necessary to stay afloat) was scored by an individual blind to the drug treatment. Time spent immobile during the swim session was scored during 4 min after the initial 2 min. Mice were intraperitoneally injected with vehicle (0.9% NaCl), l-lactate (1 g kg^−1^), d-lactate (1 g kg^−1^) or desipramine (20 mg kg^−1^) and tested 1 h later. The treatments were randomly assigned.

### Repeated open-space FST

The repeated open-space FST was performed as described previously.^20^ Swimming was carried out for 15 min per session in rat cages (43 L × 22 W × 23 H, in cm) filled with water to a depth of 15 cm at 34±1 °C. Mice swam individually for 15 min per day on 4 consecutive days before intraperitoneal administration of vehicle (0.9% NaCl), l-lactate (1 g kg^−1^), d-lactate (1 g kg^−1^) or desipramine (20 mg kg^−1^) and then two times a week until ~3 weeks had passed. Mice were allocated into the four experimental groups after the fourth day of pretest. Animals with a decreased immobility time during the pretest were excluded (two animals). Water was changed after three mice had swum to maintain a constant water temperature. Swim sessions were videotaped from above and immobility was scored by an individual blind to the drug treatment. Immobility was defined as the absence of movements, except those necessary to keep the head above water.

### Corticosterone treatment

An emulsion of corticosterone (4 mg ml^−1^) was prepared by mixing corticosterone with 2% dimethyl sulfoxide (DMSO) in sesame oil. Mice received a single subcutaneous injection of corticosterone (20 mg kg^−1^; Zhao *et al.*^21^) or vehicle (2% DMSO in sesame oil) on each of every 21 consecutive days. In addition, corticosterone-treated mice were given intraperitoneal injections of vehicle (0.9% NaCl), l-lactate (1 g kg^−1^), d-lactate (1 g kg^−1^) or desipramine (20 mg kg^−1^) daily for 21 days. The treatments were randomly assigned. At 24 h after the last injection, mice were tested in the FST (see above), tail suspension test and saccharin preference test. For the tail suspension test, mice were individually suspended by the tip of the tail with an adhesive tape on an horizontal metal bar at a height of ~34 cm. Mice were videotaped during 5 min and total immobility time was manually recorded. For the saccharin preference test, mice were singly housed in standard cages 24 h after the last drug administration. Testing was initiated by presenting two bottles (randomized to right versus left): one filled with water and the other one with a 0.02% saccharin solution. Bottles were weighed before and 48 h after saccharin access. Total consumption was calculated and data were expressed as the percentage of saccharin preference (ratio of the volume of saccharin consumed to the total volume of fluid consumed).

## Results

### Increased hippocampal lactate levels after peripheral l-lactate administration

**Figure 1:**
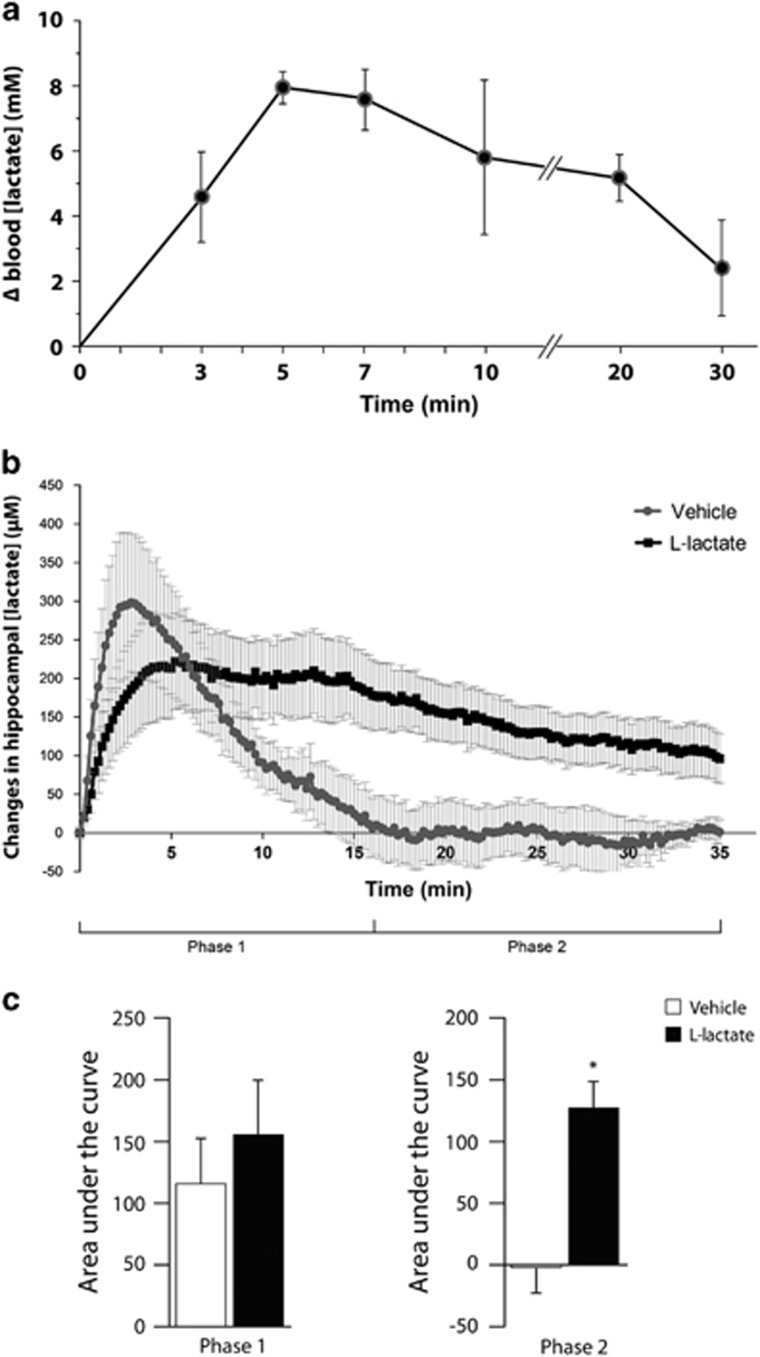
Peripheral administration of l-lactate increases extracellular l-lactate concentration in the hippocampus. (**a**) Intraperitoneal injection of l-lactate (1 g kg^−1^) increases blood lactate concentration. Data are the mean±s.e.m., *n*=5 mice per group. (**b**) Intraperitoneal l-lactate administration results in a significant elevation of hippocampal extracellular l-lactate concentration during phase 2, as measured with an l-lactate biosensor inserted within the hippocampus. Results are the mean±s.e.m., *n*=5 mice per group. (**c**) Data in (**b**) expressed as area under the curve during phases 1 and 2. *P<0.05 compared with vehicle-treated mice (Student’s *t*-test).

As an initial step in assessing the potential antidepressant effects of lactate, we established that peripheral lactate administration increased lactate concentration in the hippocampus, a key limbic structure implicated in the pathophysiology of major depression. Intraperitoneal injection of l-lactate (1 g kg^−1^), the physiological enantiomer of lactate, induced a rapid and sustained increase in blood lactate level that reached a plateau 5 min after injection and returned to near-baseline levels within 30 min (Figure 1a). The maximum increase in blood lactate concentration is within the same range of blood lactate levels measured in rodents during exercise.^22, 23^ Using an l-lactate selective biosensor inserted within the hippocampus, we found that intraperitoneal l-lactate administration resulted in a significant increase of ~200 μm in extracellular lactate concentration that slowly declined to ~100 μm within 35 min (Figure 1b). The rapid and transient elevation of extracellular lactate concentration following vehicle injection (phase 1; Figure 1b), which is not statistically different from that induced by l-lactate (phase 1; Figure 1c), is likely to be caused by injection stress as it has been shown that stress increases extracellular lactate levels in rodent hippocampus.^24, 25^ However, a significant elevation of hippocampal extracellular lactate concentration was observed during phase 2 following peripheral l-lactate administration compared with vehicle injection (Figures 1b and c).

### Antidepressant-like effects induced by acute administration of l-lactate

**Figure 2:**
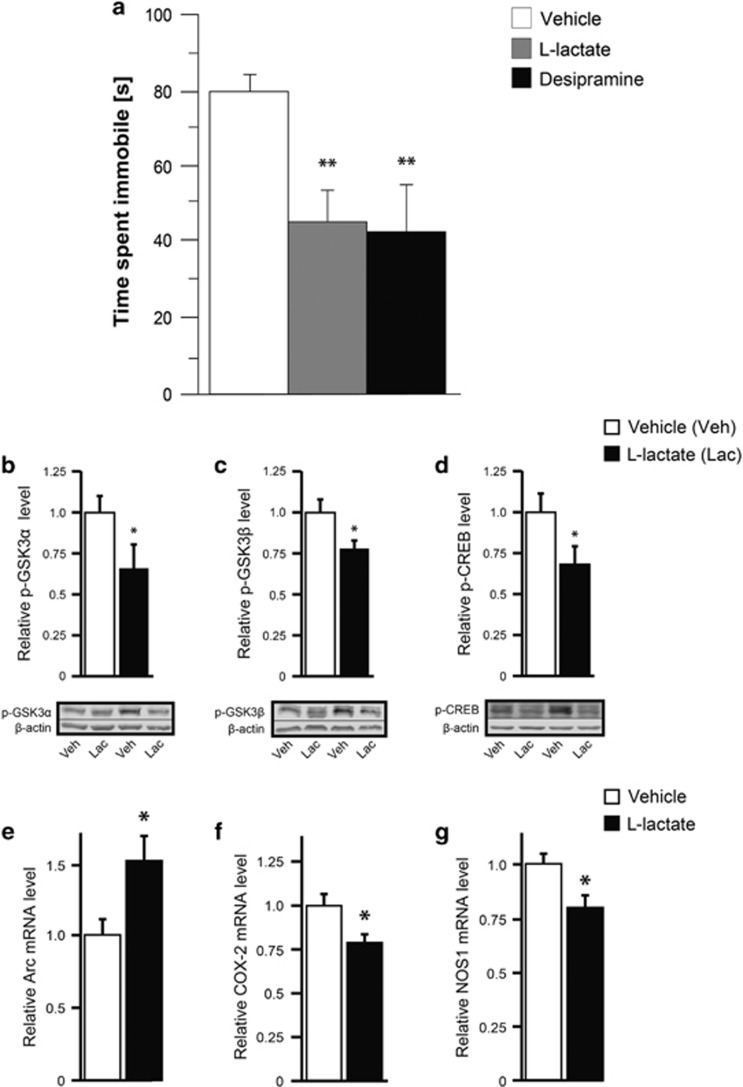
l-Lactate induces antidepressant-like effects in the forced swim test and regulates downstream signaling and target genes. (**a**) Acute peripheral administration of l-lactate produces antidepressant-like effects in the forced swim test (FST). Mice received a single intraperitoneal injection of vehicle (0.9% NaCl, *n*=17), l-lactate (1 g kg^−1^, *n*=17) or desipramine (20 mg kg^−1^, *n*=12) and were subjected to behavioral testing 1 h later. Both l-lactate and desipramine significantly reduced immobility in the FST as analyzed by one-way analysis of variance (ANOVA), followed by Tukey's *post hoc* test (F_2,4_=6.564, *P*<0.05). Data are the mean±s.e.m. **P<0.01 compared with vehicle-treated mice. (**b–d**) Phosphorylation levels of glycogen synthase kinase-3α (GSK3α), GSK3β and cAMP response element-binding protein (CREB) in the hippocampus were reduced 1 h after a single intraperitoneal injection of l-lactate, as shown by western blot analysis. Data are the mean±s.e.m., *n*=10 mice per group. *P<0.05 compared to vehicle-treated mice (Student’s *t*-test). (**e–g**) Quantitative PCR analysis revealed that a single intraperitoneal injection of l-lactate regulated the expression of Arc, cyclooxygenase-2 (COX-2) and nitric oxide synthase 1 (NOS1) mRNAs. Arc mRNA level was increased in the hippocampus of l-lactate- compared with vehicle-treated mice. COX-2 and NOS1 mRNA levels were decreased in the hippocampus of l-lactate- compared with vehicle-treated mice. Data are the mean±s.e.m. (vehicle *n*=16, l-lactate *n*=17). *P<0.05 compared with vehicle-treated mice (Student’s *t*-test).

The effect of peripheral l-lactate administration on depressive-like behavior was assessed in animal models that respond to both acute and chronic antidepressant treatment. The effect of peripheral administration of l-lactate was first measured using the FST, a well-established behavioral despair paradigm used to screen compounds for putative antidepressant activity.^26^ A single peripheral injection of l-lactate (1 g kg^−1^, intraperitoneally) reduced immobility in the FST to a similar extent as desipramine (Figure 2a), whereas the enantiomer d-lactate had no effect (Supplementary Figure 1a). Importantly, mice injected with l-lactate did not display alterations in locomotor activity and neuromuscular strength as shown in the open-field and grip strength tests (Supplementary Figure 2). Collectively, these data indicate that acute peripheral administration of l-lactate produces an antidepressant-like behavioral response in the FST.

### Regulation of signaling molecules and target genes by acute l-lactate administration

As a first step towards elucidating how l-lactate induces antidepressant-like behavior, we sought to identify the signaling pathways and target genes that are regulated in the hippocampus after a single peripheral administration of l-lactate.

Because glycogen synthase kinase-3 (GSK3) is implicated in the etiology and treatment of mood disorders,^27^ we examined the effect of l-lactate on phosphorylation level of GSK3. Phosphorylation levels of both GSK3α and GSK3β in the hippocampus were reduced 1 h after a single intraperitoneal injection of l-lactate (1 g kg^−1^) (Figures 2b and c). Accumulating evidence also supports a role of the transcription factor cAMP response element-binding protein (CREB) in the pathophysiology and treatment of depression.^28^ We therefore examined whether l-lactate regulates the phosphorylation level of CREB at Ser133. A single peripheral l-lactate administration was found to reduce phospho-CREB levels in the hippocampus 1 h after injection (Figure 2d).

To further characterize the molecular mechanisms of l-lactate action, we sought to identify target genes that are rapidly regulated by l-lactate in the hippocampus. We observed that a single peripheral injection of l-lactate upregulates the expression of Arc mRNA in the hippocampus (Figure 2e). Because previous studies suggest that inflammation increases the risk of developing major depression,^29^ we examined whether l-lactate regulated the expression of cyclooxygenase-2 (COX-2), an enzyme responsible for the formation of prostaglandins. Mice that received a single intraperitoneal injection of l-lactate showed a decreased expression of COX-2 mRNA in the hippocampus (Figure 2f). Earlier studies have shown that inhibition of nitric oxide synthase or NO production induces antidepressant-like effects in the FST.^30^ In this context, we determined whether l-lactate regulated the expression of nitric oxide synthase 1 (NOS1), the neuronal isoform of NOS. We found that NOS1 mRNA levels were decreased in the hippocampus of mice that received 1 h earlier a single peripheral administration of l-lactate (Figure 2g).

### Antidepressant-like effects produced by chronic l-lactate administration

**Figure 3:**
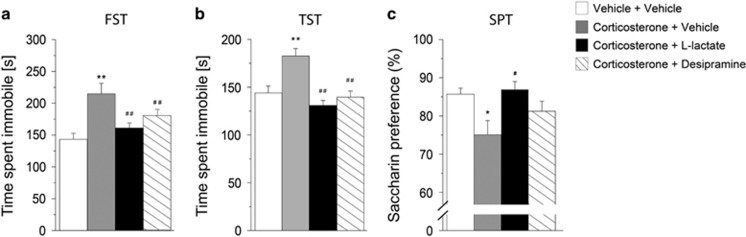
Chronic peripheral administration of l-lactate produces antidepressant-like effects in the chronic corticosterone paradigm. Mice received a single subcutaneous injection of corticosterone (20 mg kg^−1^) or vehicle (2% dimethyl sulfoxide (DMSO) in sesame oil) on each of 21 consecutive days. Corticosterone-treated mice were given intraperitoneal injections of vehicle (0.9% NaCl), l-lactate (1 g kg^−1^) or desipramine (20 mg kg^−1^) daily for 21 days. At 24 h after the last injection, mice were tested in the forced swim test (FST), tail suspension test (TST) and saccharin preference test (SPT). (**a** and **b**) Chronic administration of l-lactate and desipramine (*n*=9 and *n*=10, respectively) abolished the increased immobility induced by corticosterone treatment in the FST (F_3,45_=15.041) and TST (F_3,45_=14.253). (**c**) Chronic peripheral administration of l-lactate reversed the corticosterone-induced decrease in saccharin preference (F_3,33_=4.436). Data are the mean±s.e.m. One-way analysis of variance (ANOVA) followed by Tukey's *post hoc* test. **P<0.01 and *P<0.05 compared with vehicle + vehicle-treated mice (*n*=10); ^##^*P*<0.01 and ^#^*P*<0.05 compared with corticosterone + vehicle-treated mice (*n*=8).

We further investigated the antidepressant-like behavioral effects of l-lactate in the corticosterone model of depression. Previous studies have reported that chronic administration of corticosterone in rodents produces behavioral and neurobiological alterations that mimic symptoms and neurobiological changes associated with human depression.^31^ We therefore examined the effect of l-lactate on depressive-like behavior induced by chronic administration of corticosterone. Similarly to desipramine, we found that chronic administration of l-lactate (Figures 3a and b), but not d-lactate (Supplementary Figure 1b), abolished the increased immobility induced by corticosterone treatment in the FST and tail suspension test. In rodents, chronic corticosterone exposure induces anhedonia, a core symptom of depression.^31^ Therefore, we investigated whether l-lactate reversed the anhedonic-like behavior induced by chronic corticosterone treatment. Our data revealed that chronic peripheral administration of l-lactate reversed the corticosterone-induced decrease in saccharin consumption (Figure 3c).

**Figure 4:**
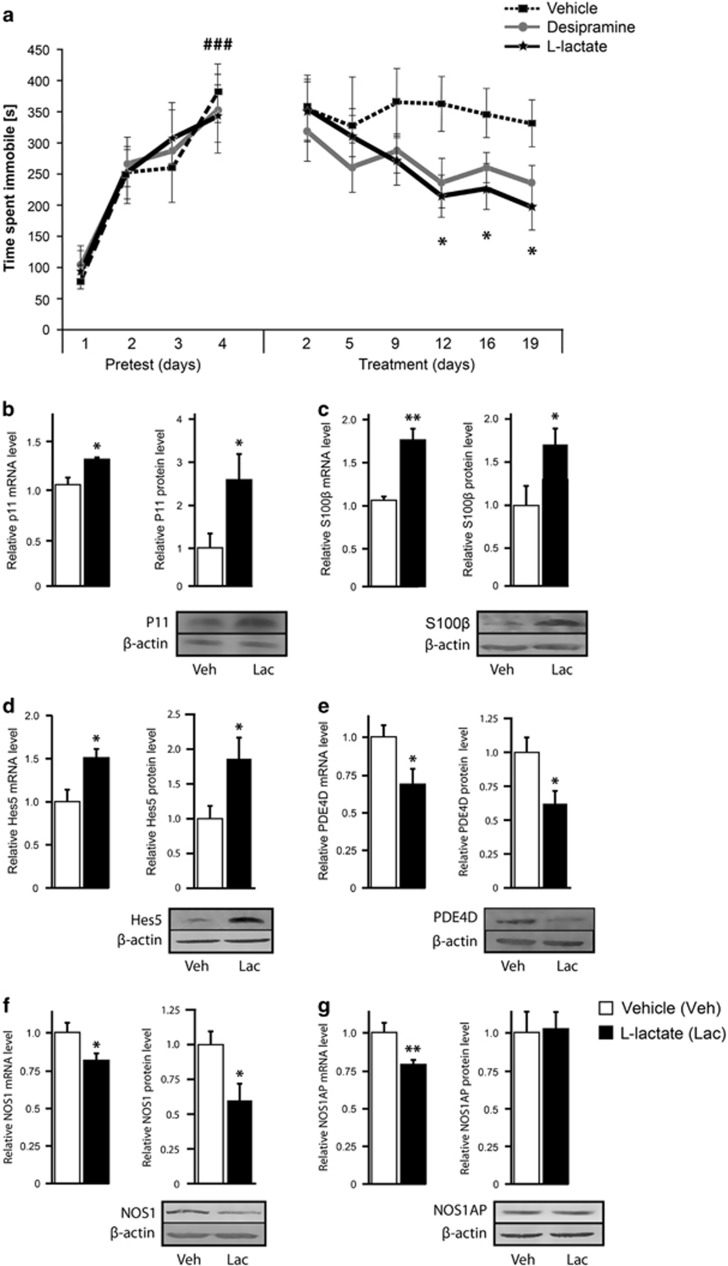
Chronic peripheral administration of l-lactate produces antidepressant-like effects in the open-space FST and regulates target gene expression. (**a**) Mice were exposed to the open-space FST according to Stone and Lin.^20^ During pretest (days 1–4), mice were subjected to four consecutive daily swim sessions. During treatment (days 1–19), mice received daily intraperitoneal administrations of vehicle (0.9% NaCl, *n*=8), l-lactate (1 g kg^−1^, *n*=10) or desipramine (20 mg kg^−1^, *n*=10) and were subjected to a swim session twice a week (at days 2, 5, 9, 12, 16 and 19). Two-ways repeated-measures analysis of variance (ANOVA) followed by Bonferroni *post hoc* test revealed a significant increase in immobility time for all groups after pretest (F_3,72_=29.377, ^###^*P*<0.001 compared with day 1). Chronic injection of l-lactate and desipramine significantly reduced immobility time as analyzed by Tukey's *post hoc* test (F_2,120_=3.961, *P<0.05 compared with vehicle-treated mice). (**b–g**) Chronic peripheral l-lactate administration regulates the expression of p11 (**b**), S100β (**c**), Hes5 (**d**), phosphodiesterase-4D (PDE4D) (**e**), nitric oxide synthase 1 (NOS1) (**f**) and NOS1AP (**g**) expression in the hippocampus of animals subjected to the open-space FST. Quantitative PCR and western blot analysis revealed that p11, S100β and Hes5 mRNA and protein levels were increased in the hippocampus of l-lactate- compared with vehicle-treated mice, whereas PDE4D and NOS1 mRNA and protein levels were decreased in the hippocampus of l-lactate- compared with vehicle-treated mice. Data are the mean±s.e.m., *n*=11 mice per group. *P<0.05, **P<0.01 compared with vehicle-treated mice (Student’s *t*-test). FST, forced swim test.

Finally, the effect of peripheral l-lactate administration on depressive-like behavior was investigated in the the open-space forced swim model of depression. This behavioral paradigm induces a chronic depression-like state that responds to chronic but not acute antidepressant treatment.^20, 32^ Daily injection of l-lactate for 3 weeks decreased the immobility time in the open-space forced swim model of depression to a similar extent as desipramine (Figure 4a). In contrast to l-lactate, chronic administration of d-lactate did not reduce the immobility time in this behavioral model of depression (Supplementary Figure 1c).

### Analysis of target genes regulated by chronic l-lactate administration

Characterization of the molecular mechanisms underlying the chronic antidepressant-like effects of l-lactate revealed that chronic peripheral administration of l-lactate regulated the expression of a specific group of genes involved in major depression and antidepressant treatment. Thus, chronic peripheral l-lactate administration increased mRNA and protein levels encoding the regulator of serotonin receptors p11,^33^ the astrocytic marker S100β^1^ and the transcription factor Hes5^34^ in the hippocampus of animals subjected to the open-space FST compared with vehicle-treated animals (Figures 4b–d).

In addition to p11, S100β and Hes5, the expression of cAMP-specific phosphodiesterase-4D (PDE4D) and NOS1 was found to be decreased by chronic peripheral administration of l-lactate both at the mRNA and protein levels in the hippocampus of animals subjected to the open-space FST compared with vehicle-treated animals (Figures 4e and f). Although NOS1AP mRNA levels were decreased by chronic l-lactate administration, NOS1AP expression at the protein level remained unaltered (Figure 4g).

## Discussion

The present set of data shows that peripheral administration of l-lactate produces antidepressant-like effects in different animal models of depression that respond to acute or chronic antidepressant treatment. In particular, chronic peripheral l-lactate administration completely reverses the corticosterone-induced anhedonia-like behavior (Figure 3c) and partially restores mobility in the open-space forced swim model of depression (Figure 4a). Importantly, these antidepressant effects of l-lactate, that are similar to those induced by desipramine, are not reproduced by the enantioner d-lactate (Supplementary Figure 1) and do not result from changes in locomotor activity and muscle strength (Supplementary Figure 2).

Acute peripheral administration of l-lactate increases hippocampal extracellular lactate concentration (Figure 1b) and regulates downstream signaling proteins (Figures 2b–d) and target genes (Figures 2e–g) in the hippocampus. The elevated hippocampal lactate concentration following acute peripheral lactate administration (Figure 1b) is consistent with previous data showing that a net uptake of lactate into the human brain is observed when arterial lactate levels increase such as during lactate infusion or intense exercise.^35, 36^

Among the genes regulated by a single peripheral injection of l-lactate (Figures 2e–g), Arc is important for synaptic function and dysregulation of Arc expression may lead to cognitive disorders.^37^ Recent data from our laboratory have shown that l-lactate increases the expression of synaptic plasticity-related genes including *Arc*, *c-fos* and *Zif268* in cortical neurons.^11^ With regard to major depression, the expression of Arc is decreased in the prefrontal cortex of depressed subjects and in the medial prefrontal cortex of mice susceptible to chronic social defeat stress.^12^ Conversely, chronic antidepressant administration and electroconvulsive stimulation upregulate Arc mRNA expression in the hippocampus and parietal cortex.^38^ In addition, administration of the fast-acting antidepressant ketamine induces Arc expression in the rat prefrontal cortex and other cortical regions.^39^ These findings support the view that the expression of Arc is downregulated in depressive-like states in human and rodents and is upregulated following antidepressant treatment. In line with this view, the increased expression of Arc in the hippocampus following a single injection of l-lactate (Figure 2e) is likely to be implicated in the acute antidepressant-like effects of l-lactate.

Previous studies indicate that inflammatory processes may be involved in patients suffering from major depression. As proinflammatory cytokines and tumor necrosis factor-α production is increased in depressed patients,^40^ anti-inflammatory treatment should reduce depressive symptoms. In this regard, treatment with the COX-2 inhibitor celecoxib decreases depressive symptoms in depressed patients.^41^ In rodents, chronic treatment with celecoxib reverses chronic unpredictable stress-induced depressive-like behavior through reduction of COX-2 expression.^42^ Collectively, these data suggest that the decreased expression of COX-2 by l-lactate in the hippocampus (Figure 2f) may contribute to the acute antidepressant actions of l-lactate.

Increasing evidence indicates that inhibition of NOS induces antidepressant-like effects.^43^ In particular, pharmacological inhibition of NOS1 produces antidepressant-like effects in the FST^30^ and chronic unpredictable mild stress.^44^ Inhibition of NOS1 expression in the hippocampus following acute (Figure 2g) and chronic administration of l-lactate (Figure 4f) is consistent with these findings and suggests that the decreased expression of NOS1 is important in mediating the acute and chronic antidepressant-like effects of l-lactate.

Previous studies have provided evidence that dysregulation of GSK3 promotes susceptibility to mood disorders and that inhibition of GSK3 activity reduces depression- and manic-like behaviors.^27^ Interestingly, inhibition of GSK3 phosphorylation by a single administration of l-lactate (Figures 2b and c) contrasts with the acute effect of the antidepressant fluoxetine that increases GSK3 phosphorylation in the prefrontal cortex and hippocampus.^45^

The transcription factor CREB is involved in the pathophysiology of depression and its treatment by antidepressants.^28^ Most studies have shown that CREB expression and activity are increased by chronic treatment with antidepressants in rodents and post-mortem human brain.^46, 47^ However, acute administration of antidepressants does not affect CREB phosphorylation in the hippocampus.^48^ Interestingly, electroconvulsive shocks, a therapy used for severely depressed patients, activates protein phosphatase 2A and reduces GSK3β and CREB phosphorylation,^49^ similarly to what was observed after a single administration of l-lactate (Figures 2c and d).

P11 was initially identified as a binding protein for 5-HTR1B, 5-HTR1D and 5-HTR4 and overexpression of p11 was shown to increase 5-HTR1B and 5-HTR4 expression at the cell surface, thereby amplifying 5-HT signaling.^33^ Levels of P11 are decreased in the anterior cingulate cortex and nucleus accumbens of depressed patients as well as in a genetic animal model of depression.^33^ Conversely, antidepressants from different classes and electroconvulsive therapy increase p11 levels in the frontal cortex and hippocampus of rodents.^33^ The increased expression of p11 by l-lactate in the hippocampus of animals subjected to the open-space FST (Figure 4b) is consistent with these studies and supports a role for p11 in the chronic antidepressant-like effects of l-lactate.

Although brain imaging and post-mortem studies have identified changes in the number and shape of specific neuronal populations in the brain of depressed patients, abundant evidence indicates that structural and functional abnormalities of astrocytes have a major role in the development of depression.^1^ In particular, human post-mortem studies have revealed alterations in the expression of astrocyte markers in different frontolimbic brain regions.^1^ Among astrocyte markers, the expression of the calcium-binding protein S100β is reduced in the ventral prefrontal cortex of depressed suicides^50^ and the density of S100β-immunopositive astrocytes is decreased in the CA1 pyramidal layer of depressed subjects.^51^ In contrast, chronic treatment with the selective serotonin reuptake inhibitor antidepressant fluoxetine increases S100β expression in the rodent hippocampus.^52, 53^ Our data showing that l-lactate administration increases S100β expression in the hippocampus of animals subjected to the open-space FST (Figure 4c) are in line with these observations, and suggest that S100β may be a mediator of the chronic antidepressant-like effects of l-lactate.

Hes5 is a downstream effector of Notch signaling, a pathway that has a central role in regulating hippocampal neurogenesis.^54, 55^ Stress, a major risk factor for depression, decreases hippocampal neurogenesis, whereas chronic antidepressant treatment normalizes neurogenesis.^56^ The increased expression of Hes5 by l-lactate (Figure 4d) in the hippocampus of animals subjected to the open-space FST suggests that stimulation of hippocampal neurogenesis may be involved in the chronic antidepressant-like effects of l-lactate.

Several lines of evidence suggest that dysregulation of cAMP-mediated signaling is involved in the pathophysiology of depression. Thus, elevation of intracellular cAMP via pharmacological inhibition of PDE4 enzymes induces antidepressant-like effects in animal models.^57, 58^ Further studies have shown that the PDE4D subtype has a pivotal role in the antidepressant-like effects of PDE4 inhibitors. Thus, mice lacking PDE4D^59^ or with reduced expression of PDE4D in the prefrontal cortex^60^ exhibit antidepressant-like behavior. The inhibitory effect of l-lactate on the expression of PDE4D in the hippocampus of animals subjected to the open-space FST (Figure 4e) is consistent with these findings and suggests that reduction of PDE4D expression may contribute to the chronic antidepressant-like effects of l-lactate.

Collectively, data regarding the characterization of the molecular mechanisms underlying the chronic antidepressant effects of l-lactate show that l-lactate acts by regulating the expression of proteins involved in 5-hydroxytryptamine receptor trafficking, astrocyte functions, neurogenesis, NO synthesis and cAMP signaling.

Taken together, these studies identify a previously unrecognized action of l-lactate by which acute and chronic peripheral administration produces antidepressant-like behavioral responses. Peripheral l-lactate injection increases hippocampal lactate levels and regulates downstream signaling molecules and target genes that may contribute to the antidepressant action of l-lactate. Further elucidation of the mechanisms underlying the antidepressant effects of l-lactate may help to identify novel therapeutic targets for the treatment of depression.
