# Erratum: Peripheral administration of lactate produces antidepressant-like effects

**DOI:** 10.1038/mp.2016.237

**Published:** 2016-12-06

**Authors:** A Carrard, M Elsayed, M Margineanu, B Boury-Jamot, L Fragnière, E M Meylan, J-M Petit, H Fiumelli, P J Magistretti, J-L Martin

**Keywords:** Depression, Pharmacology, Mechanism of action, Therapeutics

**Correction to:** *Molecular Psychiatry* advance online publication, 18 October 2016; doi:10.1038/mp.2016.179

Following publication of the above article, the authors noticed that an obsolete version of the Supplementary Information file was published. The correct version of the file accompanies this erratum online. The publisher regrets the error.

## Supplementary information

Supplementary Information (DOCX 18 kb)

