# Supplementary figures and images for: Peripheral administration of lactate produces antidepressant-like effects
Source: Mol Psychiatry. 2016 Oct 18;23(2):392–9. doi: 10.1038/mp.2016.179 (PMC5794893; doi:10.1038/mp.2016.179)

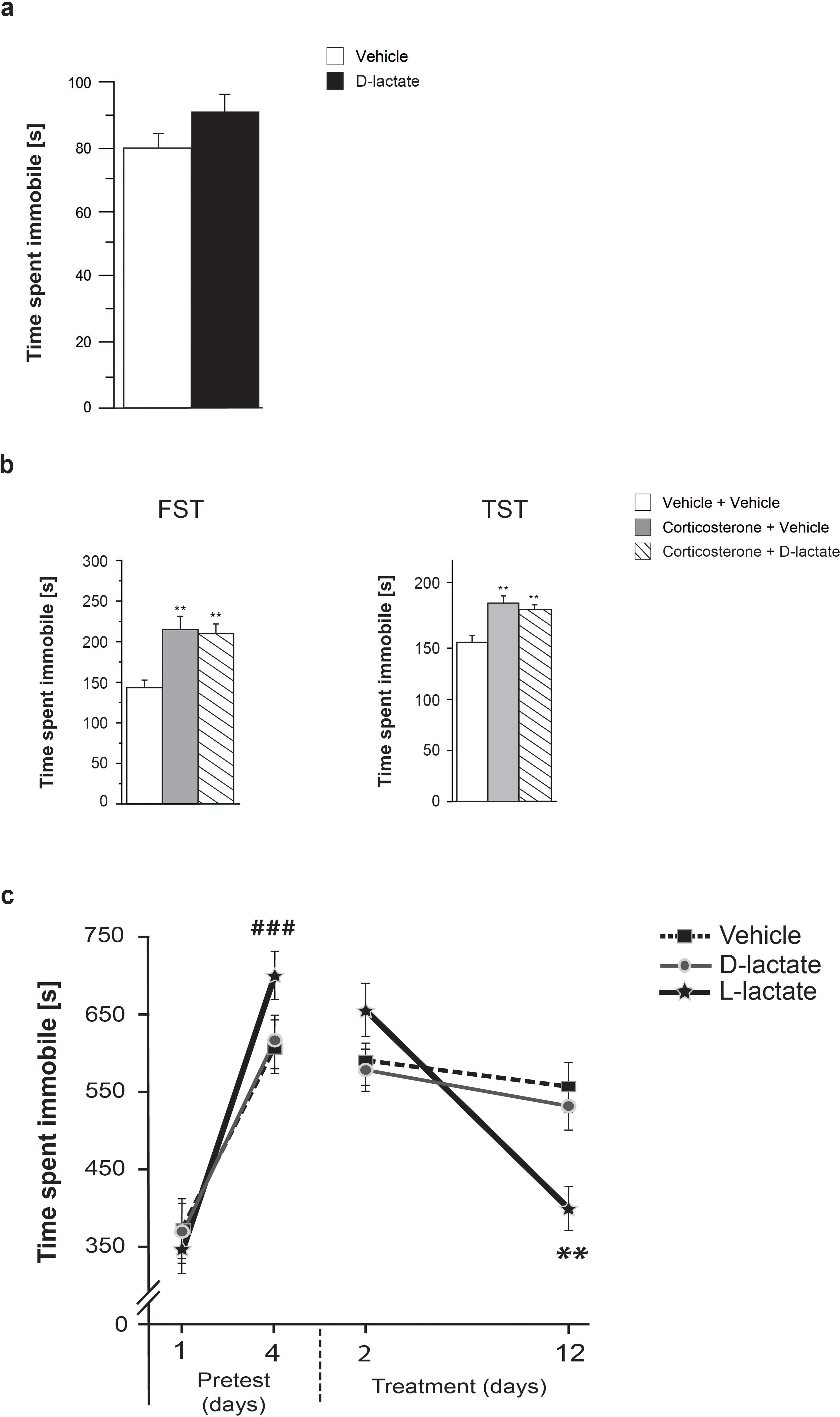

Supplement: Supplementary Figure 1 [file mp2016179x1.tif]
