# Supplementary material for: Peripheral administration of lactate produces antidepressant-like effects
Source: Mol Psychiatry. 2016 Oct 18;23(2):392–9. doi: 10.1038/mp.2016.179 (PMC5794893; doi:10.1038/mp.2016.179)
Supplement: Supplementary Table and Figure Legends [file mp2016179x4.docx]

**Figure legend for Supplementary Figure 1**

*Peripheral administration of D-lactate does not produce antidepressant-like effects in the forced swim test, chronic corticosterone paradigm and open-space forced swim test (OSFST).* **(a)** Mice received a single i.p. injection of vehicle or D-lactate and were subjected to the forced swim test. D-lactate did not reduce immobility in the forced swim test. Data are the mean ± SEM, n = 17 mice per group (Student’s *t*-test). **(b)** Mice received a single injection of corticosterone or vehicle on each of 21 consecutive days. Corticosterone-treated mice were given i.p. injections of vehicle or D-lactate daily for 21 days. One-way ANOVA followed by Tukey post-hoc test revealed that D-lactate did not reduce the increased immobility induced by corticosterone treatment in the forced swim test (FST) (F_2,18_ = 30.001, Vehicle + Vehicle n = 8, Corticosterone + Vehicle n = 5, Corticosterone + D-Lactate n = 8) and tail suspension test (TST)(F_2,17_ = 9.858, n = 7, 7 and 6, respectively). Data are the mean ± SEM. **p < 0.01 compared to vehicle + vehicle-treated mice. **(c)** Mice were subjected to the OSFST ^20^. During treatment, mice received daily i.p. administrations of vehicle, L-lactate or D-lactate and were subjected to a swim session twice a week. Two-ways repeated measures ANOVA followed by Bonferroni post-hoc test revealed a significant increase in immobility time for all groups after pretest (F_1,27_ = 65.141, ^###^p < 0.001 compared to day 1). Tukey post-hoc test revealed a significant decrease in immobility time by L-lactate (F_2,120_ = 3.961, **p < 0.01 compared to vehicle-treated mice, n = 10 mice per group) but not by D-lactate.

**Figure legend for Supplementary Figure 2**

*Acute peripheral administration of L-lactate does not affect grip strength and locomotor activity.*

**(a)** Mice received a single i.p. injection of vehicle (0.9% NaCl, n = 7), L-lactate (1 g/kg, n = 8), D-lactate (1 g/kg, n = 7) or desipramine (20 mg/kg, n = 7) and grip strength was analyzed 1, 3, 6 and 24 h later. Data were normalized to baseline levels and expressed as percent change from baseline. Two-ways repeated measures ANOVA revealed a treatment x time interaction (F_6,69_= 2.071). Tukey post-hoc analysis revealed that desipramine- but not L-lactate- or D-lactate-treated mice increased grip strength force 1 h and 3 h after drug administration. Data are the mean ± SEM. *p < 0.05 compared to vehicle-treated mice. **(b)** Mice received a single i.p. injection of vehicle (0.9% NaCl, n = 12), L-lactate (1 g/kg, n = 12), D-lactate (1 g/kg, n = 8) or desipramine (20 mg/kg, n = 12) and were tested for locomotor activity 1 h later. One-way ANOVA followed by Tukey post-hoc test revealed that, in contrast to L-lactate and D-lactate, desipramine decreased distance crossed, consistent with previous data (Tilley and Gu, JPET 327, 554-560, 2008). Data are the mean ± SEM. F_3,40_ = 10.021. **p < 0.01 compared to vehicle-treated mice.

**Figure legend for Supplementary Table 1**

List of primers used in this study for quantitative PCR analysis
