# Supplementary material for: Erratum: Peripheral administration of lactate produces antidepressant-like effects
Source: Mol Psychiatry. 2016 Dec 6;23(2):488. doi: 10.1038/mp.2016.237 (PMC5794873; doi:10.1038/mp.2016.237)
Supplement: Supplementary file 1 — Supplementary Information (DOCX 18 kb) [file 41380_2018_BFmp2016237_MOESM69_ESM.docx]

**Supplementary Information**

***Materials and methods***

***Drugs/Reagents***

Sodium L-lactate, sodium D-lactate, desipramine hydrochloride, corticosterone, sesame oil, saccharin, DMSO were from Sigma-Aldrich (Buchs, Switzerland). L-lactate, D-lactate and desipramine were dissolved in sterile saline (0.9% NaCl). Mice received L- or D-lactate at a dose of 1 g/kg and administered i.p. in a volume of 5 ml/kg; desipramine at a dose of 20 mg/kg and administered i.p. in a volume of 10 ml/kg and corticosterone at a dose of 20 mg/kg and administered s.c. in a volume of 5 ml/kg.

***Animals***

Experiments were conducted in accordance with the Swiss Federal Guidelines for Animal Experimentation and were approved by the Cantonal Veterinary Office for Animal Experimentation (Vaud, Switzerland). 8-10 week old male C57Bl/6 mice (Janvier Labs, France) were housed under a 12 h light-dark cycle at a temperature of 22℃ ± 2℃ with a relative humidity of 55% ± 10%. Mice had *ad libitum* access to water and standard rodent chow diet.

***Measurements of blood lactate concentration***

Blood lactate concentration was measured before and 3, 5, 7, 10, 20 and 30 min following i.p. injection of vehicle (0.9% NaCl) or L-lactate (1 g/kg). The treatments were randomly assigned. A drop of blood was collected with a 23-gauge needle inserted in the tail vein and lactate concentration was measured with the Lactate Pro 2 analyzer (Arkray, Japan). Mean increase in lactate concentration was obtained by subtracting the mean lactate concentration of vehicle-treated animals from lactate concentration of L-lactate-treated animals at each time point.

***In vivo determination of L-lactate concentration in the hippocampus***

Mice were anesthetized with isoflurane (4.5% for induction and 2-2.5% for maintenance), placed in a stereotaxic frame (Kopf, CA, USA) and fixed with ear bars. Cannulas for biosensor recording (Basic Guide kit number 7004, Pinnacle technology Inc., KS, USA) were implanted in the ventral part of the hippocampus (A/P -3.7 mm, ML ± 2.5 mm and DV -2.5 mm) and sealed with dental acrylic resin. Mice were allowed to recover from the surgery for one week. Prior to biosensor implantation, each microelectrode was tested initially *in vitro* to ensure proper selectivity and sensitivity for L-lactate by assessing the response of the electrode to lactate. On the experimental day, mice were placed in a recording chamber connected to a flexible tether. L-lactate biosensor probe (Pinnacle technology Inc., kit number 7004) with an integrated Ag/AgCl reference electrode was then implanted through the guide cannula in freely moving mice. Few hours after biosensor probe implantation and stabilization of the current signal; mice were i.p injected with either vehicle (0.9% NaCl), or L-lactate (1 g/kg). The treatments were randomly assigned. Drug injections were counterbalanced and a minimum of 35 min separated the injections. Then, biosensors were gently removed and post-calibration was performed *in vitro.* All lactate electrodes responded robustly to L-lactate (5.02 ± 0.96 nA/1 mM L-lactate) and calculations of the factor necessary to convert output data expressed in current (nA) to L-lactate concentration (µM) were made for each biosensor. Biosensor measurements were determined by averaging 1 sec biosensor readings over the course of each 10-sec epoch. The amperometric difference between measurements made pre- (a 5 min average baseline) and post-drug injection was calculated and converted into L-lactate concentration using the post-calibration conversion factor. Data were plotted as changes in L-lactate concentrations (µM) following injections across time expressed in min. Data are also expressed as area under the curve calculated for each of the treatments. The area under the curve was calculated using the area of the trapezoid and the following formula, (y1+y2)/2 * height, with the height being 1 sec. For each animal, the sum of all the trapezoid areas was calculated for the 35 min period and the average of the sums for each treatment group is reported. The area under the curve is expressed in two phases: phase 1, changes in L-lactate concentration during injection stress (represents the first 16 min after injection) and phase 2, changes in L-lactate concentration independent of injection stress (represents the remaining 19 min after phase 1).

***Grip strength test***

Mice were placed on a grip strength meter (Bioseb, France) to measure their neuromuscular strength. Grip strength of the front paws or of the 4 paws (both front and hind paws) was analyzed. Each mouse was tested in two sequential trials and the highest grip strength value was recorded. Measurements were made before (baseline) and after i.p. injections of vehicle (0.9% NaCl), L-lactate (1 g/kg), D-lactate (1 g/kg) and desipramine (20 mg/kg) and the effects on grip strength test were examined 1, 3, 6 and 24 h later. The treatments were randomly assigned. Data were expressed as % of change in grip force 1 h after drug injection compared to baseline.

***Locomotor activity test***

Mice were placed in a circular open field box (39 cm x 33 cm) for 30 min. Mice were videotaped and locomotor activity was measured using EthoVision Pro Video Tracking System (Noldus, Holland) in the absence of the observer. Movement was defined as spatial displacement of the body center with speed exceeding 2 cm/sec, and with threshold for no movement being 1.7 cm/sec. Mice were i.p. injected with vehicle (0.9% NaCl), L-lactate (1 g/kg), D-lactate (1 g/kg) or desipramine (20 mg/kg) and were tested for locomotor activity 1 h later. The treatments were randomly assigned.

***Western Blotting***

Western blotting was performed with rabbit anti-phospho GSK3α/β (Ser21/9)(1:1000, Cell signaling technology, #9331), rabbit anti-phospho CREB (Ser133)(1:1000, Cell signaling technology, #9198), goat anti-p11 (1:1000, R&D Systems, AF2377), rabbit anti-S100β (1:1000, Cell signaling technology, #9550), rabbit anti-HES5 (1:1000, Abcam, ab25374), rabbit anti-nNOS (1:1000, Cell signaling technology, #4231), rabbit anti-PDE4D (1:500, Santa Cruz Biotechnology, sc-25814), rabbit anti-CAPON (NOS1AP)(1:1000, Abcam, ab190686) and mouse anti-β-actin (1:1000, Abcam, ab6276) antibodies. Detection was performed using the Odyssey® infrared imaging system (LI-COR Biosciences) with anti-mouse IRDye700DX (RL610130121), anti-rabbit IRDye800 (RL611632122) and anti-goat IRDye800 (RL92532214) secondary antibodies (1:10000, Rockland). Results were quantified with Image studio lite software (Li-COR), and densitometric values were normalized to corresponding β-actin levels.

***Quantitative PCR analysis***

Mice were i.p. administered with vehicle (0.9% NaCl) or L-lactate (1 g/kg) and sacrificed 1 h after a single administration of L-lactate or 24 h after the last administration of L-lactate in mice subjected to the repeated open-space forced swim test. The hippocampi were dissected and total RNA was isolated using RNAeasy mini kit (Qiagen, CA, USA) according to the manufacturer’s instructions. 100 ng of total RNA was used for reverse transcription into first-strand cDNA with Taqman reverse transcription reagents (Applied Biosystems, CA, USA). The resulting cDNA was then amplified by real-time quantitative PCR using Power SYBR Green PCR Master Mix (Applied Biosystems) and different sets of primers. Sequences of forward and reverse primers are shown in Table 1 (Supplementary Table 1). Data were computed using the sequence detector software SDS 2.3 (Applied Biosystems) and analyzed with Excel using a macro developed by the genomic platform of Geneva University (Frontiers in Genetics, University of Geneva, Switzerland). The analysis of relative mRNA levels was performed using a delta-Ct (ΔΔCt) relative quantification model with β-actin and TBP as reference genes.

***Statistics***

Data were shown as mean ± SEM. The normal distribution and homogeneity of variances were assessed using Shapiro-Wilk and Bartlett’s tests, respectively. Sample size was determined to obtain a power of at least 0.8 using Gpower Analysis Software (v3.1.9.2 Düsseldorf University, Germany). All experiments were replicated at least twice. For the repeated open-space forced swim test, data were subjected to a two-factor repeated measures analysis of variance (ANOVA) followed by Bonferroni and Tukey post-hoc tests to confirm intra-session and intra-group differences, respectively. Bonferroni corrections were used to limit the rate of false positives in intra-session analysis. Although Tukey post-hoc test is less conservative, it allows pairwise comparisons between groups with different sample sizes and represents a good compromise for intra-group analysis. For all other analyses, student’s *t*-test or one-way ANOVA followed by Tukey post-hoc test was performed. Statistical analyses were performed with StatView 5.0 (SAS Institute, Cary, NC, USA), using an alpha level of 0.05.
